# A monograph proposing the use of canine mammary tumours as a model for the study of hereditary breast cancer susceptibility genes in humans

**DOI:** 10.1002/vms3.61

**Published:** 2017-03-21

**Authors:** Katie Goebel, Nancy D. Merner

**Affiliations:** ^1^ Department of Drug Discovery and Development Harrison School of Pharmacy Auburn University Auburn Alabama; ^2^ College of Veterinary Medicine Auburn University Auburn Alabama

**Keywords:** comparative oncology, canines, animal model, hereditary breast cancer, comparative genomics, heterogeneity, canine mammary tumours, gene discovery, next‐generation sequencing

## Abstract

Canines are excellent models for cancer studies due to their similar physiology and genomic sequence to humans, companion status and limited intra‐breed heterogeneity. Due to their affliction to mammary cancers, canines can serve as powerful genetic models of hereditary breast cancers. Variants within known human breast cancer susceptibility genes only explain a fraction of familial cases. Thus, further discovery is necessary but such efforts have been thwarted by genetic heterogeneity. Reducing heterogeneity is key, and studying isolated human populations have helped in the endeavour. An alternative is to study dog pedigrees, since artificial selection has resulted in extreme homogeneity. Identifying the genetic predisposition to canine mammary tumours can translate to human discoveries – a strategy currently underutilized. To explore this potential, we reviewed published canine mammary tumour genetic studies and proposed benefits of next generation sequencing canine cohorts to facilitate moving beyond incremental advances.

## Background

As a component of the One Health Initiative (One Health Initiative, 2016), comparative oncology capitalizes on the fact that naturally occurring tumours in companion animals are excellent models of human cancer and can accelerate genetic, pathological and pharmaceutical discoveries (Davis & Ostrander 2014). Cancer is the leading cause of death in aged dogs, and is often spontaneous and similar to human cancers in its clinical presentation and pathophysiology (Davis & Ostrander 2014; Liu *et al*. 2014; Schiffman & Breen 2015). As loved members of human families, dogs are generally kept until old age, and are second only to humans in the level of health care that they receive (Rowell *et al*. 2011). Furthermore, canines age five to eight times faster than humans, providing an expedited model of disease onset and progression (Rowell *et al*. 2011). These facts, considered in conjunction with the large population of pet dogs in the United States (about 70 million) (Schiffman & Breen 2015), as well as the number of concerned breeders, establish dogs as the best‐known and most attainable mammalian model of human cancers (Rowell *et al*. 2011; Ostrander & Franklin 2012; Davis & Ostrander 2014; Liu *et al*. 2014; Schiffman & Breen 2015).

Although scientists are still deciphering the exact date and aetiology of canine domestication from wolves (Vonholdt *et al*. 2010), it is well known that the creation of modern dog breeds is a relatively recent phenomenon that occurred approximately 200 years ago and represents a significant evolutionary bottleneck (Sutter & Ostrander 2004; Rivera & von Euler 2011; Rowell *et al*. 2011; Ostrander & Franklin 2012). The artificial selection of dog breeds is largely based on human preferences and current trends, which is likely to continue. Through generations of linebreeding and influence of the popular sire effect (breeding one champion stud dog widely), most dog breeds were established from a small number of founders. The genetic characteristics of those founders are therefore currently overrepresented in the breed population (Patterson 2000). These give each breed its distinctive morphologic and behavioural traits, as well as predispositions to genetic diseases (Ostrander & Franklin 2012). In 2000, 370 canine genetic disorders were recognized and >50% were breed‐specific (Patterson 2000). This makes sense because strict pedigree barriers prevent interbreeding, suggesting that each breed represents an isolated population with potentially its own unique mutations and resultant diseases (Patterson 2000; Melin *et al*. 2016).

Breed or kennel‐based studies represent the human familial approach to disease gene discovery with the additional resource of well‐documented and large pedigrees, making them extremely attractive for genetic linkage studies. Generally, obtaining large, informative and properly ascertained human families is difficult, and to achieve the necessary statistical strength, families must often be grouped together for analysis (Chandler *et al*. 2016). This is not an ideal approach for genetically heterogeneous disorders, especially in outbred human populations. Thus, similar to studying humans from geographically isolated or founder populations, studying dog breeds provides a strategy to reduce genetic heterogeneity, since ancestral mutations occur at higher frequencies and contribute towards breed‐specific diseases (Ostrander & Franklin 2012). Highlighting the power of canine linkage analysis, in 2000, a single dog pedigree stemming from a German Shepherd sire that had litters with 6 different females was studied to identify the genetic locus of RCND (renal cystadenocarcinoma and nodular dermatofibrosis), a rare inherited cancer syndrome (Jonasdottir *et al*. 2000). A year later, the equivalent human cancer syndrome, Birt‐Hogg‐Dube syndrome, was mapped (Schmidt *et al*. 2001), and identification of the orthologous disease gene in both species immediately followed (Nickerson *et al*. 2002; Lingaas *et al*. 2003). Since that time, genetic mapping of canine disorders has contributed to major medical advancements. A short list of human disease genes that were first mapped in dogs includes those for narcolepsy, copper toxicosis, neuronal ceroid lipofuscinosis and ichthyosis (Ostrander & Franklin 2012).

In 2005, the first version of the canine genome was published (Lindblad‐Toh *et al*. 2005), which truly set the stage for comparative genomics. Since that time, both genome‐wide association studies (GWAS) and whole exome sequencing (WES) studies have been carried out to identify disease genes in canines that also explain the equivalent trait in humans, for example (Sloan *et al*. 2011; Grall *et al*. 2012). Compared to human GWAS studies, smaller dog cohorts can be studied (Davis & Ostrander 2014). It is possible that only 50 000 single nucleotide polymorphisms (SNPs) and 200 dogs would be needed to determine disease loci (Ostrander & Franklin 2012), compared to the thousands of cases and millions of SNPs needed in a human experiment (Michailidou *et al*. 2013). Not surprisingly, several medical research institutes have launched programs/projects promoting the use of purebred dogs as human cancer models, including two institutes of the National Institutes of Health (NIH), the National Cancer Institute (NCI) and the National Human Genome Research Institute (NHGRI), as well as the Broad Institute. The NCI developed the Comparative Oncology Program in 2003; the NHGRI has an internal research branch devoted to Cancer Genetics and Comparative Genomics; the Broad Institute initiated the Dog Disease Mapping Project. Furthermore, related extramural funding opportunities are available through the NIH as well as national breed registration organizations such as the American Kennel Club. With such current interest in this research area as well as the recent design and optimization of canine exome enrichment kits (Broeckx *et al*. 2015), even more translatable discoveries should unfold. This includes discoveries that enhance our knowledge of hereditary breast cancer (BC) genetics.

BC is prevalent in both humans and canines. It affects one in eight American women and, referred to as canine mammary tumours (CMTs) in the veterinary field, is the most common neoplasia in intact female dogs. In both species, identified risk factors include age, obesity, hormonal effects and genetics (Rivera & von Euler 2011; ACS, 2014; Melin *et al*. 2016). A true understanding of the genetic contributions to BC has yet to be fully grasped due to the heterogeneity of the disease and studied human cohorts, and its apparent polygenic inheritance (Chandler *et al*. 2016). The aims of this review are to argue for the use of the dog as a hereditary BC model and to summarize CMT genetic variant analyses reported to date. Regrettably, CMT genetic studies have been limited.

### Canines as models of hereditary BC

#### Barriers to hereditary BC susceptibility gene discovery

Hereditary BC is characterized by a strong family history, early ages of onset (less than 45 years of age), bilateral presentations, affected males, as well as the appearance of other associated cancers, such as ovarian and prostate cancer, in the family (Berliner & Fay 2007). The genetic variant(s), or germline mutations, that segregate in hereditary BC families and increase risk of the disease, have an apparent autosomal dominant pattern of inheritance. Overall, genetic variants that increase risk of developing BC are divided into three broad groups of penetrance/risk, including high, moderate and low penetrant variants. Genes that harbour such variants are referred to as BC susceptibility genes (Chandler *et al*. 2016).

High and moderate risk variants are rare (normally defined as having a minor allele frequency (MAF) of less than 1%), and confer lifetime risks of over 50% and between 25 and 50%, respectively (Chandler *et al*. 2016). Over 35 hereditary BC susceptibility genes have been suggested to contain such risk variants, however, not all have sufficient statistical data confirming risk (Easton *et al*. 2015). A high or moderate penetrant variant that segregates in a family can be the major contributing allele that explains the increased familial risk, yet less than 30% of BC‐affected individuals with a personal or family history of the disease have such a variant in a currently reported BC susceptibility gene (Chandler *et al*. 2016). Two of these genes, *BRCA1* (Miki *et al*. 1994) and *BRCA2* (Wooster *et al*. 1995), were discovered in the 1990s and have been well‐documented as harbouring high and moderate risk variants. These are the most frequently mutated BC susceptibility genes to date, and the variants within them convey lifetime BC risks of 55–85% and 35–60%, respectively (Chandler *et al*. 2016). Together, *BRCA1/2* mutations explain 15% of hereditary BC cases (Shiovitz & Korde 2015). Although extensively studied, in total, these findings leave over 70% of hereditary BC cases genetically unsolved.

Low penetrant BC variants are generally common (have a MAF >1%, which are referred to as SNPs) and, individually, only increase risk of disease by approximately 1.5‐fold compared to the average American women (Chandler *et al*. 2016). Over 70 BC‐associated SNPs have been reported, mainly identified through GWAS (Michailidou *et al*. 2013); and recently, researchers have tried to quantify the contributions of multiple low penetrant variants, theorizing that these could additively contribute to BC risk. Polygenic risk scores (PRS) were calculated and could explain up to a 3‐fold increased risk (for women in the highest percentile of PRS); however, women diagnosed with BC under the age of 40 years and/or with a family history of the disease were in the lowest PRS percentile (Mavaddat *et al*. 2015). This suggests that more rare and penetrant genetic variants likely explain the occurrence of hereditary BCs.

Since 2011, a number of WES studies have been carried out to identify additional rare variants that increase risk of hereditary BC (Chandler *et al*. 2016). WES is a next generation sequencing (NGS) approach (Shendure & Ji 2008) that targets the exome (all the coding regions in the genome) and was initially reported to aid in disease gene discovery efforts in 2010 (Ng *et al*. 2010a, 2010b). The hereditary BC WES studies that produced the most statistically significant data involved studying isolated/founder human populations (Chandler *et al*. 2016), suggesting that reducing heterogeneity is key to BC susceptibility gene discovery.

#### CMTs as a genetic model of human BCs

CMTs represent very practical models for human BCs since they share clinical, molecular, histological and epidemiological characteristics (Rivera & von Euler 2011; Visan *et al*. 2016). For instance in both species, females are primarily affected, but some male cases have been reported. Additionally, both species often develop mammary tumours as they age, with the average age of incidence hovering at about 10 years for most breeds (Rivera *et al*. 2009). Furthermore, hormonal influence is another commonality between BC and CMTs, and veterinarians and clinical researchers have extensively recorded the benefit of ovariohysterectomy (spaying) of female dogs to prevent CMT development (Egenvall *et al*. 2005; Jitpean & Hagman 2012; Liu *et al*. 2014). Ultimately, similar to human BCs, CMTs take a variety of histopathological forms, and about 50% of cases are malignant (Rivera *et al*. 2009; ACS, 2014; Melin *et al*. 2016). This, however, also highlights the importance of noting CMT and BC differences. For example, unlike in humans, it is common for CMTs to occur at multiple sites in an affected dog with varying histology within and between the different tumours. Plus, certain histological characteristics, such as myoepithelial cell proliferation, occur more often in CMTs than human BC (Visan *et al*. 2016). It is important to note that all animal models of disease present differences from the human condition, but, overall, the spontaneous and heterogeneous nature of CMTs best mimic human BC risk and development making the domestic dog a valuable genetic model of human BC.

Unlike for other forms of cancer, researchers do not agree upon which breeds have the greatest mammary cancer susceptibility or prevalence, as geographic and breed popularity contributions are confounding, and sources that report primary data are few (Sutter & Ostrander 2004; Egenvall *et al*. 2005; Borge *et al*. 2011, 2013; Jitpean & Hagman 2012; Davis & Ostrander 2014). One suspected “high‐risk” breed mentioned by multiple studies is the English Springer Spaniel (ESS) from Sweden. Average age of CMT onset in the ESS breed is approximately 7 years, mirroring the early‐onset observed in human familial BC cases (Rivera *et al*. 2009; Borge *et al*. 2013; Melin *et al*. 2016). While this breed may present an attractive model, it should be noted that spaying of dogs is uncommon in Sweden (Egenvall *et al*. 2005; Jitpean & Hagman 2012), so care must be taken to discern genetic from hormonal stimuli.

The genetics of CMTs is understudied, despite the fact that genetic analysis of CMTs began twenty years ago (Szabo *et al*. 1996; Van Leeuwen *et al*. 1996). It has long been documented that high‐risk BC susceptibility genes are well conserved between humans and dogs. In 1996, (Szabo *et al*. 1996) categorized known human *BRCA1* missense mutations, and sequenced dog and mouse DNA (complementary and/or genomic) to investigate homology between species. Several of the mutations were located at shared amino acid residues across species, and a handful of those were also within conserved domains. Notably, *BRCA1* sequence homology data was supportive of the use of a dog as a comparative genomics model: the researchers reported an 84% dog‐human nucleotide similarity versus 72% mouse‐human, and 73.8% dog‐human amino acid identity versus 55.9% mouse‐human identity (Szabo *et al*. 1996). The amino and carboxyl termini of the BRCA1 protein were highly conserved among all three species, especially including the zinc finger motif. The central portion of the BRCA1 protein is the most divergent among species, but closer in dog‐human (85% identity) than in mouse‐human (74%) (Szabo *et al*. 1996). Such conservation and similarity of these gene and protein segments is indicative of functional significance in both species.

#### CMT genetic studies – detecting germline risk variants

With the introduction of NGS, it is now well documented that tumours are tremendously genetically diverse regarding acquired (somatic) mutations (Russnes *et al*. 2011). Identifying such mutations is a hot area of research that began over two decades ago in both humans (Hollstein *et al*. 1994) and canines (Devilee *et al*. 1994). In fact, the initial focus of CMT genetic research involved identifying somatic mutations (Van Leeuwen *et al*. 1996), and a recent WES study has begun to advance our knowledge in this area (Liu *et al*. 2014). Regarding hereditary cancers, discovering the germline (inherited) genetic variants that drive cancer development before somatic mutations accumulate is an area that needs research focus. However, to date, just over a handful of publications have focused on identifying inherited genetic risk factors of CMT.

The first germline mutation associated with CMT was reported in the *p53* tumour suppressor gene, which has long been classified as an important cancer catalyst in humans (Veldhoen *et al*. 1999). Matched normal and cancerous mammary tissues were selected from a cohort of 10 dogs, and a wealth of clinical information was available (age, breed, intact/spayed status, tumour histopathology and veterinarian's prognosis). One patient, a five‐year‐old Boxer, had two distinct germline mutations reported, each on a different parental chromosome. This included a large deletion of exons three through seven, and a P69L substitution in exon three – both predicted to be pathogenic (Table 1) (Veldhoen *et al*. 1999). These appear to be the only canine germline *p53* mutations reported to date. It will be interesting to determine the true contribution of *p53* mutations towards CMT genetics upon additional and larger sequencing studies.

**Table 1:** Reported canine germline coding variants in orthologs of known or candidate BC susceptibility genes

Gene	rs§	Nomenclature			Reference
		Genomic*	mRNA†	Protein‡	
*BRCA1*	rs397510981	chr9:g.19985060	c.723A>G§	p.K241K	(Enginler *et al*. 2014)
	rs397512112	chr9:g.19985075	c.738T>A§	p.T246T	(Enginler *et al*. 2014)
	rs397509570	chr9:g.19988291	c.3954G>A	p.S1318S	(Borge *et al*. 2011)
*BRCA2*	rs23250374	chr25:g.7787056	c.428A>G∥	p.H143R	(Borge *et al*. 2011)
	rs23244160	chr25:g.7775050	c.2401A>C	p.K801Q	(Borge *et al*. 2011)
	–	–	c.2414G>A***	p.R805L	(Hsu *et al*. 2010)
	rs397511123	chr25:g.7768691_7768693	c.6918_6920delGTT	p.L2307del	(Borge *et al*. 2011; Enginler *et al*. 2014)
	rs23255542	chr25:g.7768681	c.6930C>T§	p.F2310F	(Rowell *et al*. 2011; Enginler *et al*. 2014)
	rs397509895	chr25:g.7747589	c.9138A>G¶	p.L3046L	(Enginler *et al*. 2014)
	–	chr25:g.7747332	c.9308A>G	p.K3103R	(Borge *et al*. 2011; Enginler *et al*. 2014)
	rs397510884	chr25:g.7735440	c.9968G>A	p.S3323N	(Enginler *et al*. 2014)
	rs853007536	chr25:g.7735654	c.9995_9996insAAA**	p.M3332delinsIK	(Yoshikawa *et al*. 2005; Borge *et al*. 2011; Enginler *et al*. 2014)
*BRIP*	rs397511741	chr9:g.34983082	c.3029G>A	p.R1010H	(Borge *et al*. 2011)
	rs397512960	chr9:g.34983223	c.3170C>T	p.P1057L	(Borge *et al*. 2011)
*CDH1*	–	chr5:g.80784440_80784442	c.387_389delCCA	p.H129del	(Borge *et al*. 2011)
	rs397512866	chr5:g.80776897	c.945C>T	p.S315S	(Borge *et al*. 2011)
*EGFR*	rs9206306	chr18:g.5996046	c.677G>A	p.R226Q	(Borge *et al*. 2011)
	rs397513721	chr18:g.5996076	c.707C>T∥	p.P236L	(Borge *et al*. 2011)
*HER2 (ERBB2)*	rs397510212	chr9:g.22773443	c.1105A>G∥	p.K369E	(Borge *et al*. 2011)
	rs24616607	chr9:g.22770524	c.1575G>C	p.P525P	(Borge *et al*. 2011)
	rs24537329	chr9:g.22770473	c.1626A>G	p.E542E	(Borge *et al*. 2011)
	rs24537331	chr9:g.22770288	c.1728C>T	p.C576C	(Borge *et al*. 2011)
	rs397510076	chr9:g.22766833	c.1905G>A	p.A635A	(Borge *et al*. 2011)
	rs397512599	chr9:g.22763063	c.2769T>C	p.Y923Y	(Borge *et al*. 2011)
	rs397512289	chr9:g.22761328	c.3486G>A	p.P1162P	(Borge *et al*. 2011)
	rs397510013	chr9:g.22761055	c.3759C>T	p.Y1253Y	(Borge *et al*. 2011)
*ESR1*	rs21960513	chr1:g.42131190	c.627T>C	p.F209F	(Borge *et al*. 2011)
	rs397512038	chr1:g.42208686	c.979A>G	p.I327V	(Borge *et al*. 2011)
	rs397512133	chr1:g.42364093	c.1578G>A	p.L526L	(Borge *et al*. 2011)
*PTEN*	–	chr26:g.37910150	c.975C>T	p.L325L	(Borge *et al*. 2011)
*TP53*	–	chr5:g.32564669	c.206C>T∥	p.P69L	(Veldhoen *et al*. 1999)
	–	chr5:g.32564760_32562912	Germline deletion of exons 3‐7∥		(Veldhoen *et al*. 1999)

*Genome build: Broad CanFam3.1/canFam3 (Dog Assembly. Sept. 2011). ^†^ *Nucleotide accession numbers: BRCA1*: NM_001013416.1*, BRCA2*: NM_001006653.4*, BRIP*: XM_847556.4*, CDH1*: NM_001287125.1*, EGFR*: ENSCAFT00000005575.3, *HER2*: NM_001003217.2*, ESR1*: NM_001286958.1, *PTEN*: NM_001003192, *TP53*: NM_000546.5. ^‡^ *Protein accession numbers:* BRCA1: NP_001013434, BRCA2: NP_001006654, BRIP: ENSCAFT00000045493.2, CDH1: NP_001274054.1, EGFR: ENSCAFT00000005575.3, HER2: NP_001003217, ESR1: NP_001273887.1, PTEN: NP_001003192, TP53: NP_001003210. ^§^variants found only in CMT‐affected dogs. ^¶^claimed to be associated with CMT. ^∥^predicted to be pathogenic in respective papers. **variant was initially suspected pathogenic but is now considered neutral. ***variant is named as reported in Hsu *et al*.; due to lack of information presented, locating this variant within NM_001006653 and Broad CanFam3.1/canFam3 was not possible.

**Figure 1:**
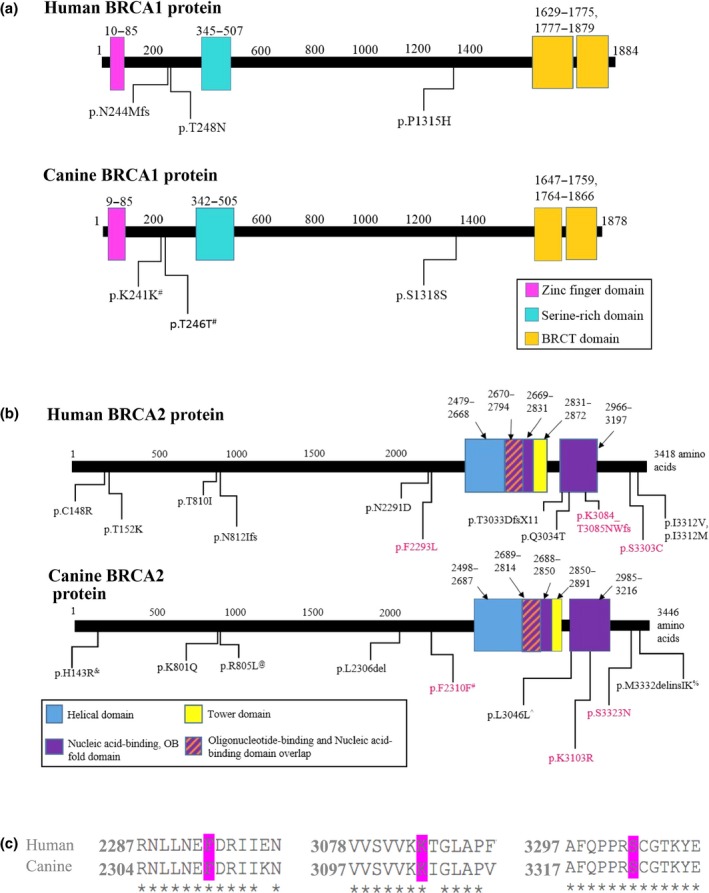
Illustrations of human and canine BRCA1 and BRCA2 proteins. All known canine coding variants in BRCA1 (Panel A) and BRCA2 (Panel B) proteins are noted on the diagram. Known human variants reported in Breast Cancer Information Core (BIC) within 2 amino acids of the conserved position of a canine variant were noted as well; the conserved human residues and locations were determined through a protein alignment. Hot pink text indicates conserved canine and human amino acid residues with variants; see Panel C for amino acid alignment. ^#^variants found only in CMT‐affected dogs;^^^claimed to be associated with CMT; ^&^predicted to be pathogenic in respective papers; and ^%^initially suspected pathogenic but is now considered neutral, and ^@^named as reported in Hsu *et al*. due to lack of information presented, which limited locating variant within NM_001006653 and Broad CanFam3.1/canFam3. *Human protein accession numbers:* BRCA1: NP_009231, BRCA2: NP_000050; *Canine protein accession numbers:* BRCA1: NP_001013434, BRCA2: NP_001006654

In the mid‐late 1990s and early 2000s, the canine sequences of *BRCA1* (Szabo *et al*. 1996) and *BRCA2* (Bignell *et al*. 1997; Ochiai *et al*. 2001) were generated to obtain a reference sequence that would aid in the analysis of CMT susceptibility. A number of variations were reported, but their identification in normal canine mammary or other non‐tumoral tissues(Szabo *et al*. 1996; Bignell *et al*. 1997; Ochiai *et al*. 2001) left no connection to disease. Benign and pathogenic variants exist (Richards *et al*. 2015), and well‐designed studies are required to associate variants with a disease. The first presumed canine *BRCA2* cancer‐risk variant was reported in 2005 by Yoshikawa *et al*. (2005) (p.M3332IK; Table 1 and Fig. 1), the same group that previously published the complete canine *BRCA2* sequence (Ochiai *et al*. 2001; Yoshikawa *et al*. 2005). However, this study involved the analysis of blood‐extracted DNA samples from 21 tumour‐free dogs with no clinical or breed data made available. Furthermore, the insertion was detected in 17 of the 21 dogs; 10 were homozygous and seven were heterozygous (Yoshikawa *et al*. 2005). The authors claimed that the variant altered a predicted nuclear localization segment within the C‐terminus of the canine BRCA2 protein, and noted that the same sequence in the human BRCA2 protein harboured two missense variants (p.I3312V and p.I3312M; Fig. 1). Noteworthy, these two human variants are reported to be of unknown significance in the Breast Cancer Information Core (BIC) database. Since BRCA2 was already known to interact with Rad51 at the extreme C‐terminus and predicted to play a role in the handover of Rad51 to DNA substrates, hybridization assays were carried out to note interaction differences with and without the variant. Ultimately, the presence of p.M3332IK reported a slightly stronger BRCA2/Rad51 interaction, which could disturb the transfer of Rad51 to its substrate, leading to the authors' pathogenicity prediction (Yoshikawa *et al*. 2005). Despite the fact that this variant has been reported in additional sequencing studies that aimed to identify germline CMT‐risk variants (Table 1), no associations could be claimed (Borge *et al*. 2011; Enginler *et al*. 2014); in fact, a subsequent paper by Yoshikawa *et al*. (2012) reported the variant to be neutral.

Rivera *et al*. (2009) were the first to report an association between CMT and variants in *BRCA1* and *BRCA2*. They studied ESSs from Sweden, where 36% of the population is affected, by carrying out a case–control study involving 212 CMT cases and 143 CMT‐unaffected controls. Ten canine orthologs of genes either known or predicted to increase risk to human BC were selected for study, and four to nine common SNPs were selected per gene, totalizing 63 genotyped SNPs. Overall, statistically significant associations were detected for one variant in *BRCA2* and two in *BRCA1*. Interestingly, odds ratios of ~4 were calculated for both genes. Thus, the authors suggested that a common CMT‐predisposing allele exists in both *BRCA1* and *BRCA2* in this ESS population (Rivera *et al*. 2009). The associated SNPs were intronic or appeared non‐functional, so the quest to identify the exact risk variants remains open.

Several Sanger sequencing studies that aimed to detect germline CMT‐risk variants have been carried out. Firstly, it should be noted that in 2010 a study by Hsu *et al*. (2010) sequenced exon 11 of canine *BRCA2* using DNA extracted from 11 CMTs and four randomly collected normal mammary tissues. The main objective was to identify genetic variants in the CMT samples associated with different tumour histological type and prognosis. Since matched normal mammary tissues were not sequenced in parallel, this approach prevented the true classification of the CMT reported variants as somatic or germline. However, two assumed somatic variants were identified as hot spots that may have prognostic potential since they were detected in the majority of the studied CMTs, and specifically, in all dogs with stage V mammary carcinosarcomas and rapid disease progression. Interestingly, one of those two hot spot alleles, an arginine at amino acid position 805 (p.R805, corresponding to a guanine at mRNA position 2414), is reported in two canine *BRCA2* reference sequences (AB043895 and Z75664). The authors did report p.R805L (c.2414 G>A) in the normal mammary tissues that were sequenced as a reference in their study, which differed from the previously reported reference sequences, presumably classifying it as a germline *BRCA2* variation (Table 1). Determining whether a germline allele at this position is associated with CMT requires further investigation.

In 2011, Borge *et al*. sequenced eleven genes (*BRCA1, BRCA2, BRIP1, CDH1, CHEK2, EGFR, ESR1, HER2, PTEN, STK11,* and *TP53*) in 32 dogs from eight separate breeds (Borge *et al*. 2011). The breeds were evenly divided into “high risk” (Boxer, Cocker Spaniel, English Springer Spaniel, and Standard Poodle) and “low risk” (Bernese Mountain Dog, Cavalier King Charles Spaniel, Shetland Sheepdog and St. Bernard) groups to simulate a case–control study. Blood for DNA extraction was obtained from four randomly selected dogs from each breed and the authors did not have knowledge of clinical CMT status. Twenty‐five coding variants were reported; this included nine non‐synonymous, 13 synonymous, two deletions and one insertion (Table 1) (Borge *et al*. 2011). No statistically significant allele frequency differences were recorded between high and low risk breeds. However, three of the detected non‐synonymous variants were predicted to be damaging, *BRCA2* p.H143R, *EGFR* p.P236L and *HER2* p.K369E (Table 1; Fig. 1). The deletions (one each in *BRCA2* and *CDH1*) and insertion (in *BRCA2*) affected 3 bps each (Table 1; Fig. 1); thus, no frameshifting mutations were identified. The *BRCA2* insertion was the same as reported by Yoshikawa and colleagues (Table 1; Fig. 1) (Yoshikawa *et al*. 2005; Borge *et al*. 2011). Overall, the authors provided the first comprehensive list of coding variants in cancer‐associated genes and highlighted potentially pathogenic variants that they suggested are likely associated with CMT. Subsequently, the same group carried out a follow‐up case–control genotyping study that aimed to further investigate those probable associations (Borge *et al*. 2013). Common SNPs within all the genes listed above and sequenced in the previous study (Borge *et al*. 2011), minus *TP53*, which was previously investigated by Rivera *et al*. (2009) in a similar study, were genotyped in a case–control cohort of ESS and in a second group of dogs that were either at high or low risk of CMT. Ultimately, several SNPs of significance within the *ESR1* gene were identified (Borge *et al*. 2013). Although broad in scope, these efforts are marred by the assumption that breeds have conferred differing propensities for CMTs, a yet‐to‐be‐proven assertion.

Confirmed CMT susceptibility genes *BRCA1* and *BRCA2* (Fig. 1) had thus far returned goading variant profiles. Thus, the next case–control study surveyed female dogs (25 with and 10 without a CMT diagnosis) from a variety of breeds for genetic differences specifically in *BRCA1* and *BRCA2* (Enginler *et al*. 2014). Clinical information such as age, age at spaying or intact status, tumour histopathology, body weight and breed were all noted. Using DNA extracted from the blood of affected and unaffected cohorts, selected regions of *BRCA1* and *BRCA2* were sequenced, and *BRCA2* p.L3046L was shown to be significantly associated with CMTs (Table 1; Fig. 1). Several other variants were found only in the CMT‐affected dogs and not in controls, but were not statistically significant. These included *BRCA1* p.K241K and p.T246T, and *BRCA2* p.F2310F (Table 1; Fig. 1) (Enginler *et al*. 2014); the latter was also identified in a previous study (Borge *et al*. 2011). Additional investigation of these variants and application of a similar but larger experimental design could bolster future efforts.

The most recent CMT genetics publication that aimed to identify inherited CMT‐risk factors described the first CMT GWAS (Melin *et al*. 2016). The study cohort was comprised of only ESS dogs, but interestingly, dogs from Swedish as well as Norwegian and British populations were examined. Blood and buccal swab samples were acquired from client‐owned dogs at veterinary clinics over several years, along with pertinent clinical data. A total of 332 ESSs (188 cases and 144 controls) were genotyped for over 130 000 SNPs. Ultimately, genome wide significance was obtained for one SNP on chromosome 11; seven other SNPs on chromosomes 11 and 27 were nominally associated, identifying three potential CMT‐risk loci (one on chromosome 11 and two on chromosome 27) (Melin *et al*. 2016). Further analysis identified an associated haplotype on chromosome 11 that encompassed *CDK5RAP2*, which encodes a cyclin‐dependent kinase involved in cell cycle regulation. Moreover, *LACRT* and *SLC38A4*, which encode a glycoprotein involved in tear secretion and an amino acid transporter, respectively, were suggested candidate genes for the two chromosome 27 loci.

Overall, pitfalls of the current CMT genetic studies include sequencing small segments of already‐known high‐penetrant BC genes, and using cohorts where CMT‐affection status of the sequenced individuals and their predecessors remains unknown. Furthermore, when on a quest to identify breed‐specific risk variants, focusing on only one specific breed (ESS) or, on the complete opposite end of the spectrum, designing studies with small cohorts of multiple breeds, is not efficient. The currently published studies have specifically associated variants in *BRCA1, BRCA2* (Rivera *et al*. 2009; Enginler *et al*. 2014), and *ESR1* (Borge *et al*. 2013) with CMT risk. Drawing from known BC susceptibility and general DNA repair/maintenance genes, a host of potential CMT genes‐ *BRIP, CDH1, ERBB2* (also called *HER2*)*, PTEN, STK11* and *TP53*‐ are suspected (Borge *et al*. 2011), but confirmatory case–control experiments have not been conducted. This assumption provides a springboard for CMT‐variant discovery, but should not limit future and more unbiased BC/CMT gene discovery investigation (Chandler *et al*. 2016).

#### Future directions

With little known about the complete list of genes responsible for both BC and CMTs, applications of NGS to canine cohorts seems like a logical step to facilitate novel susceptibility gene discovery. Although breed predispositions are currently unclear, a simple experimental design utilizing cases within a single breed and maximally unrelated controls from the same breed could be implemented to ascertain probable predispositions. An in depth pedigree analysis would aid in the success of this approach and identify a probable CMT inheritance pattern. Overall, this method would reduce sample size to a manageable, yet statistically significant, number (Davis & Ostrander 2014; Broeckx *et al*. 2016). Applying NGS technologies towards cohorts such as these would both confirm suggested associations of previously identified coding variants and unearth novel variants involved in the disease, including variants in regulatory elements. While WES is currently the most attractive and attainable NGS method due to cost and other constraints, analysing the entire canine genome through whole genome sequencing (WGS) is worthwhile since disease‐predisposing variants are not necessarily solely located in the coding DNA (Davis & Ostrander 2014; Melin *et al*. 2016). During these studies, ascertainment of clinical data including age, sex and tumour type are invaluable and could be individually tested for association with newly identified CMT‐risk variants.

## Conclusions

Heterogeneity of the human genome has allowed our species to fight and prevail against a suite of illnesses throughout the ages, but it poses an unshakable obstacle in our advances against cancer. Embracing the use of dogs and their simplified genetic structure will likely help us overcome this barrier. Whether the end goal is to improve the health of the human or of the canine patient, in this new era of personalized medicine and comparative genomics, it is remiss to ignore the resources provided by the history of dog breeding, the sequencing of the canine genome, and latest WES and WGS technologies.

## Declarations

### Consent for publication

Not applicable.

### Availability of data and material

Breast Cancer Information Core (https://research.nhgri.nih.gov/bic/)

dbSNP (http://www.ncbi.nlm.nih.gov/snp)

Dog Disease Mapping Project (http://www.broadinstitute.org/what-is-broad/areas-focus/project-spotlight/dog-disease-mapping-project-dogdna-studying-domesticated-do)

ExPASy Bioinformatics Resource Portal SIM Protein Alignment Tool (http://web.expasy.org/sim/).

## Sources of funding

2016 Veterinary Summer Scholars Program at Auburn University, College of Veterinary Medicine (fellowship awarded to KG); American Association of Colleges of Pharmacy (AACP) New Investigator Award (2016), AURIC Seed Grant (2015–2016), AURIC Seed Grant (2016–2017), Auburn University Competitive Outreach Scholarship Grant (2016), Auburn University Innovative Research grant through the Internal Grant Program (2016–2018) (to NDM).

## Conflict of interest

The authors declare that they have no conflicts of interests.

## Ethics statement

No ethical approval was required as it is a review article.

## Authors' contributions

NDM provided background information and was key in directing literature review efforts. NDM also contributed to the manuscript outline and revisions. KG compiled the variant data and wrote the first draft of the manuscript. Figures and tables were products of a collaborative effort from both NDM and KG. All authors read and approved the final manuscript.
